# Retroperitoneal Versus Transperitoneal Robot Assisted Partial Nephrectomy: A Prospective Controlled Non‐Randomized Single Centre Study Non‐Inferiority Design

**DOI:** 10.1002/rcs.70077

**Published:** 2025-05-27

**Authors:** Rene Mager, Igor Tsaur, Thomas Höfner, Mohamed Kamal Gheith, Gregor Duwe, Maximilian Haack, Jonathan Azar, Brahim Aboulmaouahib, Stefanie Ziewers, Peter Sparwasser, Lisa Frey, Anita Thomas, Axel Haferkamp

**Affiliations:** ^1^ Department of Urology and Paediatric Urology University Medical Center Mainz Mainz Germany; ^2^ Institute of Medical Biostatistics, Epidemiology and Informatics University Medical Center Mainz Mainz Germany

**Keywords:** pentafecta, retroperitoneal, robot assisted partial nephrectomy, transperitoneal, trifecta

## Abstract

**Background:**

The value of the retroperitoneal (R‐RAPN) compared with the conventional transperitoneal (T‐RAPN) approach in robot‐assisted partial nephrectomy has not been finally clarified. The current work's objective was to prospectively investigate R‐RAPN versus T‐RAPN.

**Methods:**

The study was designed as a prospective, controlled, non‐randomized study with a non‐inferiority design. The primary endpoint was Trifecta achievement. The sample size calculation required 141 T‐RAPN and 94 R‐RAPN.

**Results:**

When the recruitment target of 141 was reached in the T‐RAPN arm, only 34 R‐RAPN had been performed, so the study was terminated early. Trifecta as the main outcome parameter was achieved in 82% of the R‐RAPN and 76% of the T‐RAPN groups, so no sign for inferiority could be detected (*p* = 0.6).

**Conclusions:**

In this prospective study, there was no evidence of inferiority of R‐RAPN compared to T‐RAPN for the Trifecta endpoint. R‐RAPN may be an individually advantageous alternative to T‐RAPN for selected patients.

**Trial Registration:**

The study was registered in the German Clinical Trials Register (DRKS00028619).

## 1 Introduction

Robot‐assisted surgery for localised kidney cancer has become widely accepted in urological surgery. Since no real randomized controlled trial comparing robot versus open surgery has ever been conducted, the level of evidence for robot‐assisted surgery does not exceed 2a [1]. In this way, in a current large and comprehensive meta‐analysis, only two of 34 included studies were of prospective design [2]. Since its early days, robot‐assisted partial nephrectomy (RAPN) has been performed using a transperitoneal access [3]. Parallel to numerous advancements in robotic surgery during the following years, the transperitoneal approach (T‐RAPN) suggesting improved access to posterior and complex tumours has been established [4]. Again, evidence for the retroperitoneal approach (R‐RAPN) is limited because only one prospective non‐randomized study has been available to be included in the latest pooled analysis addressing this issue [5]. Furthermore, in this important prospective study by Tanaka, only 10 of 26 patients underwent R‐RAPN [6]. Together with 2251 R‐RAPN and 3989 from 16 retrospective studies in this pooled analysis R‐RAPN demonstrated lower estimated blood loss, shorter operative time and shorter length of stay, while the rates of significant complications did not differ between R‐RAPN and T‐RAPN groups [5]. Since there is an obvious lack of prospective data supporting T‐RAPN, the current work aimed to strengthen the evidence for T‐RAPN with a prospectively designed trial.

## 2 Methods

### 2.1 Patients

Eligible patients were 18 years of age or older, with previously untreated localised kidney tumour scheduled for robot assisted partial nephrectomy. Exclusion criteria were recurrent tumour with previously ipsilateral surgical treatment.

### 2.2 Trial Design

**FIGURE 1:**
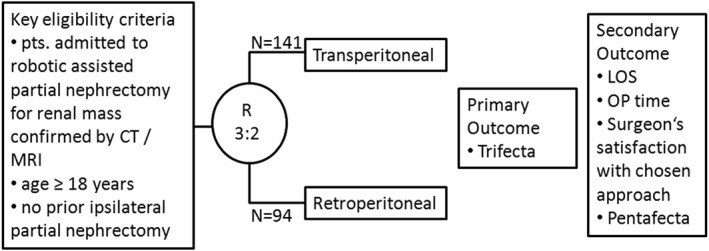
Trial design.

This was an open label, prospective, controlled and not randomized trial (Figure 1). The decision against randomisation was made for the following reasons. First, R‐RAPN has been suggested to be beneficial mainly for posterior tumours, thus randomized groups of R‐RAPN and T‐RAPN would not necessarily represent patients in the real world. Second, patients would probably refuse to participate when they are informed that their surgeon would be compelled by trial design to operate with an approach the surgeon might not be convinced to suit best with the patient's individual case characteristics. Therefore, the decision about the surgical approach was up to the operating surgeon. This decision was monitored by a questionnaire: For each case, the surgeon wrote down preoperatively the reason for the choice (estimated greater convenience, estimated accessibility, estimated blood loss, estimated lower risk of adjacent organs' injury) and the case experience (< 50 or ≥ 50 cases) and explained postoperatively if he was satisfied with the pre‐operative decision or not. After surgery a 1 year follow‐up period completed the study.

### 2.3 Transperitoneal and Retroperitoneal Access

The operations were conducted under general anaesthesia. The da Vinci Xi system (Intuitive Surgical) was used for all operations. Intravenous antibiotic prophylaxis was administered in a single shot manner 30 min before trocar placement. Patient positioning and trocar placement of the T‐RAPN group followed traditional protocols [7]. The R‐RAPN technique was performed as described by the working group of the Vattikuti Urology Institute and by Socarrás et al. [8, 9, 10].

### 2.4 Outcome Measures

The primary outcome measure was Trifecta achievement. The trifecta was defined according to Khalifeh et al. [11] as no intraoperative or postoperative complications, negative surgical margins and warm ischaemic time ≤ 25 min. Secondary outcome measures comprised common perioperative parameters such as operative time, estimated blood loss, ischaemia time, intraoperative difficulties, conversion rates, and surgeons' questionnaire answers. Postoperative outcome variables were Pentafecta achievement, 1‐year overall survival, 1‐year disease free survival, operation associated morbidity at 1‐year follow‐up, length of stay, operative time, postoperative haemoglobin drop and the rise in creatinine.

### 2.5 Ethical Statement

This trial was approved by the local ethics committee of Rhineland‐Palatinate (#2020‐15336). It was conducted according to Good Clinical Practice guidelines. All the patients provided written informed consent that was based on the Declaration of Helsinki principles. The study was registered in the German Clinical Trials Register (DRKS00028619). A data and safety monitoring committee reviewed efficacy and safety.

### 2.6 Statistical Analysis

This study was planned as a non‐inferiority trial for the primary outcome parameter Trifecta achievement. The Farrington‐Manning score test for proportion difference was used. T‐RAPN served as the benchmark approach. With the current body of literature, the authors estimated common Trifecta rates of approximately 70% for both approaches. Significance was set to *α =* 0.05. Inferiority was defined if R‐RAPN had 15% less Trifecta achievement compared with T‐RAPN. Based on previous RAPN at our centre, a case allocation of 2:3 was assumed for R‐RAPN compared to T‐RAPN. Thus, a case number of 94 r‐rapns and 141 T‐RAPN was demanded to achieve *β =* 0.8. The analyses of secondary outcome measures were only exploratory.

## 3 Results

When the recruitment of the T‐RAPN group was completed, only 34 patients were enroled in the R‐RAPN group. With respect to the slow recruitment of the R‐RAPN cases, the study's steering committee decided to early terminate the trial. As a consequence, the power to detect a significant Trifecta inferiority diminished to *β =* 0.45.

The patients' baseline characteristics are presented in Table 1. There were significant trends towards lower SPARE scores, posterior position of the tumour, and greater surgeon's experience for R‐RAPN compared with T‐RAPN.

**TABLE 1:** Baseline characteristics.

Variable	R‐RAPN	T‐RAPN	*p* value
	*N* = 34	*N* = 141	
Age, years (IQR)	66 (59, 73)	65 (57, 74)	0.7
Male/Female, *n*/*n*	21/13	96/45	0.6
Left/Right kidney, *n*/*n*	19/15	70/71	0.6
Clinical T stage, *n*			
T1a	30 (88)	124 (88)	0.9
T1b	4 (12)	16 (11)	
T2a	0	1 (1)	
SPARE score	1 (0, 2)	1 (0, 4)	0.04
SPARE risk			
Low (0–3)	29 (85)	100 71)	0.2
Intermediate (4–7)	5 (15)	40 (28)	
High (8–10)	0 (0)	1 (1)	
Posterior position, *n* (%)	23 (68)	65 (47)	0.04
Crea (mg/dL),	0.9 (0.79, 1.04)	0.95 (0.78, 1.09)	0.6
eGFR (mL/min)	83 (65, 96)	83 (67, 94)	0.9
Hb (g/dL)	14.6 (13.3, 15.5)	14.6 (13.4, 15.6)	0.6
CCI			
0–4	22		
> 4	12	7863	0.4
Prior abdominal surgery, *n* (%)	19 (56)	87 (62)	0.7
Surgeon's experience in RAPN, *n*			
< 50 cases	5 (15)	52 (37)	0.02
≥ 50 cases	29 (85)	89 (63)	

*Note:* Continuous variables are presented as median and interquartile range and were tested by Mann Whitney *U* test. Categorical variables were tested using Chi square or Fisher's exact test.

Abbreviations: CCI = Charlson comorbidity index, eGR = estimated glomerular filtration rate, SPARE = The Simplified PADUA REnal nephrometry system [12].

The analyses of perioperative outcomes revealed a significantly shorter OR time, lower EBL and less intraoperative difficulties for R‐RAPN, while warm ischaemic time and conversion to open surgery demonstrated no differences between groups (Table 2). Postoperative outcomes are provided in Table 3. Trifecta as the main outcome parameter was achieved in 82% of the R‐RAPN and 76% of the T‐RAPN groups, so no sign for inferiority of R‐RAPN could be detected (*p* = 0.6). Apart from a shorter length of stay for R‐RAPN, secondary outcome measures demonstrated no significant difference between T‐RAPN and R‐RAPN.

**TABLE 2:** Perioperative outcome.

Outcome parameter	R‐RAPN	T‐RAPN	*p* value
	*N* = 34	*N* = 141	
Operative time, min (IQR)	126 (107, 158)	142 (119, 180)	0.035
EBL, ml (IQR)	150 (150, 263)	300 (150, 450)	0.001
Ischaemia time, min (IQR)	10 (6, 14)	11 (6, 15)	0.4
Intraoperative ultrasonography, *n* (%)	1 (3)	13 (9)	0.7
Intraoperative difficulties, *n* (%)	4 (12)	39 (28)	0.03
‐ Adherent perinephric fat	2 (6)	18 (13)	0.4
‐ Adhesiolysis	0	9 (6)	0.3
‐ Complex renal hilum	2 (6)	12 (8)	0.9
Conversions, *n* (%)	3 (9)	8 (6)	0.4
‐ Open partial nephrectomy, *n*	3 (9)	5 (4)	0.4
‐ Open nephrectomy, *n*	0	2 (1)	1.0
Robot assisted nephrectomy, *n*	0	1 (1)	1.0
Surgeons' reasons for their choice			
‐ Estimated greater convenience	6	73	< 0.001
‐ Estimated better accessibility	29	73	< 0.001
‐ Estimated lower blood loss	0	22	0.008
‐ Estimated lower risk of adjacent organs' injury	0	6	0.6
Surgeons' satisfaction with their choice	100%	89%	0.04

*Note:* Continuous variables are presented as median and interquartile range (IQR) and were tested by Mann Whitney *U* test. Categorical variables were tested using Chi square or Fisher's exact test.

Abbreviation: EBL = estimated blood loss.

**TABLE 3:** Main and secondary postoperative outcomes.

	R‐RAPN	T‐RAPN	*p* value
	*N* = 34	*N* = 141	
Main outcome			
Trifecta, *n* (%)	28 (82)	107 (76)	0.6
‐ Negative surgical margins	34 (100)	141 (100)	
‐ WIT ≤ 25 min	34 (100)	141(100)	
‐ No intra‐ and postop. complications	28 (82)	107 (76)	
Secondary outcome measures			
Postoperative complications, *n* (%)	4 (12)	35 (25)	0.2
‐ Clavien Dindo Grade I	1 (3)	2 (1)	
‐ Clavien Dindo Grade II	3 (9)	24 (17)	
‐ Clavien Dindo Grade IIIa	0	8 (6)	
‐ Clavien Dindo Grade IIIb	0	1 (1)	
Transfusion rate, *n* (%)	0	8 (6)	0.3
Length of stay, d	6 (5, 7)	6 (6, 8)	0.04
Postoperative eGFR, mL/min	81 (52, 90)	73 (60, 91)	0.6
ΔeGFR, mL/min	−6 (1, 17)	−5 (2, −13)	0.3
Postoperative haemoglobin, g/dL	11.5 (10.3, 12.7)	11.6 (10.4, 12.7)	0.9
Δhaemoglobin, g/dL	−2.7 (−1.8, −3.5)	−2.7 (−1.8, −3.6)	1.0
Histology			
‐ Clear cell RCC	15 (44)	60 (43)	1.0
‐ Papillary RCC	9 (26)	26 (18)	0.9
‐ Chromophobe RCC	2 (6)	7 (5)	1.0
‐ Other malign	0	2 (1)	1.0
‐ Angiomyolipoma	3 (9)	12 (9)	1.0
‐ Oncocytoma	5 (15)	25 (18)	0.9
‐ Other benign	0	8 (6)	0.3
Follow‐up adherence at 1 year, *n* (%)	27 (79)	100 (71)	0.4
Pentafecta *n*/*N* (%)	11/23 (48)	43/75 (57)	0.6
Op. assoc. morbidity at 1y‐fu, *n*/*N* (%)	26/27 (96)	92/100 (92)	0.7
Disease free at 1y‐fu, *n*/*N* (%)	26/27 (96)	96/99 (97)	1.0
Mortality at 1y‐fu	0/27 (0)	1/100 (1)	1.0
Imaging modality at 1y‐fu			
‐ None	5 (19)	28 (28)	0.5
‐ Abdominal ultrasound	2 (7)	7 (7)	1.0
‐ Abdominal computed tomography	15 (56)	54 (54)	1.0
‐ Abdominal MRI	4 (15)	14 (14)	1.0
‐ Chest computed tomography	2 (7)	10 (10)	1.0

*Note:* Continuous variables are presented as median and interquartile range (IQR) and were tested by Mann Whitney *U* test. Categorical variables were tested using Chi square or Fisher's exact test.

Abbreviations: 1y‐fu = one year follow‐up, *d* = days, eGFR = estimated glomerular filtration rate, MRI = magnetic resonance imaging, *n* = number, RCC = renal cell carcinoma.

## 4 Discussion

This trial represents the largest prospective and controlled study comparing T‐RAPN with R‐RAPN so far. Regarding the main outcome measure Trifecta achievement, which is a well‐established binary variable derived from negative surgical margins, warm ischaemic time and complications, this study revealed no sign of inferiority for R‐RAPN against the benchmark of T‐RAPN. This is in line with results from a large body of retrospective data [12, 13, 14, 15, 16, 17, 18, 19, 20, 21, 22] and one small prospective observational study [6]. The rates of conversions to open surgery or to nephrectomy were in the range of previously reported series. In the same way, the current work demonstrated equal complication rates [6, 12, 13, 14, 15, 19, 20, 21, 22, 23].

As the groups were formed by surgeons' choice of operative approach and not by randomisation, significant differences between T‐RAPN and R‐RAPN at baseline with regard to higher tumour complexity (SPARE score), fewer posterior position and fewer surgeons experience for T‐RAPN cases were to be expected. Several published series have demonstrated the association of R‐RAPN with posterior tumour position [6, 12, 14, 18, 21, 22, 24]. Surprisingly, there was no significant difference in cases with prior abdominal surgery although advantages to perform R‐RAPN in these patients have been proposed [17, 25] and therefore were assumed to influence the surgeons' decision of operative access. Although the influence of the surgeons' experience on the decision for or against R‐RAPN has been discussed by several authors [20, 21, 24, 26], this was the first work to prove that performing R‐RAPN is significantly associated with greater experience. Again, in line with the available literature, operative time [12, 14, 16, 19, 22, 25, 27], estimated blood loss [15, 19, 21, 25, 27] and length of hospital stay [13, 22, 28] were significantly lower for R‐RAPN compared with T‐RAPN. With respect to the relatively long LOS for both R‐RAPN and T‐RAPN compared with the aforementioned studies, the authors assume a systemic influence by the lower limit of stay required by the national reimbursement regularities.

The occurrence of intraoperative difficulties is rarely reported in the available literature. The current work demonstrated a significantly higher rate of reported difficulties during T‐RAPN, which might be influenced by the group's higher SPARE score and lower case load of surgeons' experience. Although statistically nonsignificant, the numeric trend towards the preferential treatment of more complex tumours preferentially by T‐RAPN has also been reported by Arora et al. and Choo et al. [12, 15].

This work uniquely informs about the surgeons' rationale to decide for or against R‐RAPN. Interestingly, surgeons justified their choice for the T‐RAPN approach significantly more often with expected greater convenience, better accessibility and lower blood loss but at the end were significantly less satisfied with T‐RAPN compared to R‐RAPN. These findings might indicate on the one hand the surgeons' persisting greater familiarity with the T‐RAPN access and on the other hand a lower confidence in their operative ability and in the educational grading of the whole operation room staff to safely master the R‐RAPN approach.

Although the aforementioned advantages for selected R‐RAPN cases versus T‐RAPN are significant, promise benefits for the patients and have been reliably demonstrated by many authors, there is obviously no telling argument for R‐RAPN against T‐RAPN in the perception of urologic surgeons. One reason for this might be the fact, that the experienced surgeon, who is trained to handle with the retroperitoneal access feels equally skilled to complete a case with a dorsal tumour and prior abdominal surgery without surgically relevant differences in outcome by T‐RAPN [20]. Since R‐RAPN represents a more sophisticated approach with regard to educational grading of surgeons and nursing staff, the availability of all resources necessary to master the R‐RAPN procedure might be a limiting factor. Summed up, these infrastructural dynamics and clinical observations might have led to the weak accrual of R‐RAPN cases in this study. Only few available R‐RAPN studies provide 1‐year follow‐up or Pentafecta data. Based on the patients' follow‐up adherence of 73% at 1 year, the current work demonstrated no significant difference of Pentafecta, operation associated morbidity, disease free status and mortalitiy between R‐RAPN and T‐RAPN. Equal efficacy of Pentafecta achievement, GFR preservation or disease free survival after R‐RAPN and T‐RAPN have been demonstrated by Choi et al., Strooup et al., Dell Oglio et al. and Carbonara et al. in their retrospective comparative studies [14, 18, 20, 21].

Several limitations have to be mentioned. The recruitment of R‐RAPN cases did not reach the planned numbers, so statistical power to detect inferiority was diminished. It is important to understand, that the surgeons were free to decide the approach they were convinced of in a certain patient. The aforementioned factors might have driven their decisions towards T‐RAPN. A randomized design could have excluded this shift towards surgeons' familiarity but could also have resulted in a high drop‐out rate due to protocol violations. Obviously conceptualization and conduction of randomized controlled study designs in robot assisted surgery remain challenging. The lost to follow‐up rate was relatively high, so Pentafecta achievement rates might lack robustness. The significantly higher surgeons' experience in the R‐RAPN group might have favoured its outcome measures by decreasing the likelihood for inferior outcome compared with the less experienced surgeons performing the possibly superior transperitoneal approach. Since all retrospective series published so far might harbour this risk for bias, the outcome quality of R‐RAPN might be overestimated in general. In contrast, it is important to know, that there were no significant differences between R‐RAPN and T‐RAPN outcomes in the only prospective comparative trial, where differences of surgeons experience were excluded by the fact, that Tanaka et al. reported their initial RAPN case series, so there was no previous experience with any RAPN access at all [6]. In the current work, there was no patients' questionnaire to investigate the patients' expectations and demands on robotic assisted kidney surgery. Thus, it can only be hypothesized, that the ability to offer a kidney tumour patient on the basis of the individual computed tomography scan and individual medical history two different operative approaches during the pre‐operative outpatient visit might increase the patients' confidence in the state of the art quality of their urologic surgeons.

## 5 Conclusions

This prospective controlled open label non‐inferiority trial of R‐RAPN versus T‐RAPN revealed no sign of inferiority of R‐RAPN with respect to Trifecta achievement as the primary outcome parameter. Of the secondary outcomes operative time, estimated blood loss, length of hospital stay and surgeons' satisfaction with the chosen approach offered significant advantages for R‐RAPN. Thus, the retroperitoneal access harbours clinical benefit for those patients, selected for R‐RAPN by experienced robotic surgeons. Since R‐RAPN recruitment did not reach the demanded case numbers, the results are underpowered. Nevertheless, this study adds further insight into the topic of RAPN.

## Author Contributions

**Rene Mager:** supervision, data collection, writing – original draft. **Igor Tsaur:** data collection. **Thomas Höfner:** data collection. **Mohamed Kamal Gheith:** writing – review and editing. **Gregor Duwe:** data collection. **Maximilian Haack:** data collection. **Jonathan Azar:** data collection, writing – original draft. **Brahim Aboulmaouahib:** supervision, writing – review and editing. **Stefanie Ziewers:** data collection. **Peter Sparwasser:** data collection. **Lisa Frey:** data collection. **Anita Thomas:** writing – review and editing. **Axel Haferkamp:** supervision, data collection, writing – review and editing. All authors read the final manuscript and agreed with authorship and publication.

## Ethics Statement

This trial was approved by the local ethics committee. It was conducted according to Good Clinical Practice guidelines. All the patients provided written informed consent that was based on the Declaration of Helsinki principles.

## Conflicts of Interest

The authors declare no conflicts of interest.

## Permission to Reproduce Material From Other Sources

No materials from other sources were used.

## Data Availability

The data that support the findings of this study are available on request from the corresponding author. The data are not publicly available due to privacy or ethical restrictions.
